# Single-use flexible bronchoscopes vs traditional reusable flexible bronchoscopes: a prospective controlled study

**DOI:** 10.1186/s12890-023-02478-5

**Published:** 2023-06-09

**Authors:** Shuzhen He, Lihua Xie, Jianming Liu, Lijun Zou

**Affiliations:** Department of Pulmonary and Critical Care Medicine, The Third Xiangya Hospital of Central South University, Changsha, China

**Keywords:** Single use bronchoscopes, Traditional reusable bronchoscopes, Bronchoalveolar Lavage, Bronchoscope biopsy

## Abstract

**Background:**

Single-use flexible bronchoscopes(SFB) eliminate the risk of bronchoscopy-related infection compared with traditional reusable flexible bronchoscopes(RFB). At present, there is no comparative study between SFB and RFB in the aspects of biopsy and interventional therapy. This study aims to explore whether SFB can perform complex bronchoscopic procedures such as transbronchial biopsies just like RFB.

**Methods:**

We conducted a prospective controlled study. A total of 45 patients who required bronchoscopic biopsy in our hospital from June 2022 to December 2022 were enrolled. The patients were divided into the SFB group and the RFB group, and routine bronchoscopy, bronchoalveolar lavage, and biopsy were performed respectively. Data on the time of routine bronchoscopy, the recovery rate of bronchoalveolar lavage fluid(BALF), biopsy time, and bleeding volume were collected. Then we used the two-sample t-test and the χ^2^ test to assess the performance differences between SFB and RFB. We also designed a questionnaire to compare the performance between SFB and RFB by different bronchoscope operators.

**Results:**

The routine examination time of SFB and RFB was 3.40 ± 0.50 min and 3.55 ± 0.42 min, respectively. There was no significant difference between the two groups (*P* = 0.308). The recovery rate of BALF was (46.56 ± 8.22) % in the SFB group and (47.00 ± 8.07) in the RFB group, without a significant difference between the two groups(*P* = 0.863). The biopsy time was similar(4.67 ± 0.51 min VS 4.57 ± 0.45 min) in both groups, with no significant difference(*P* = 0.512). The positive biopsy rate was 100% in both groups, with no significant difference. Overall, the bronchoscope operators were generally satisfied with SFB.

**Conclusion:**

SFBs are non-inferior to RFBs in routine bronchoscopy, bronchoalveolar lavage, and biopsy. It is suggested that SFBs have a wider clinical application.

## Background

With the development of medical technology, traditional reusable flexible bronchoscopes (RFBs) plays a crucial role in the diagnosis and treatment of tracheobronchial and pulmonary diseases. However, it is worth noting that there are three significant limitations of RFBs: risk of cross-infection [1, 2], high maintenance costs [3], and long waiting times for decontamination [4]. Especially in the COVID-19 global pandemic, it is particularly important to reduce the risk of cross-infection. Previously, researchers reported that RFBs may cause cross-contamination [5-7], with an infection risk of 2.8% [8]. Several studies have also shown that common pathogens causing bronchoscopy-related infections include Pseudomonas aeruginosa, Klebsiella pneumonia, Escherichia coli, and so on [7, 9-11]. It can increase the risk of cross-infection and death, and increase the medical cost.

Since Ambu company first invented a single-use flexible bronchoscope (SFB) in 2009 [12, 13], Some of these problems have been solved. The greatest benefit of SFBs is the avoidance of cross-infection, especially in the intensive care unit (ICU) [4, 14]. Nosocomial infections and cross-infection are very common in ICU and the SFBs will play a substantial role, especially in patients who are mechanically ventilated, immunosuppressed, or have infectious diseases. Therefore, for protecting the healthcare staff the SFB is undoubtedly a safer option for immunocompromised patients or to avoid cross-infection with infectious diseases [4, 15]. To date, SFBs have been used in ICU or the operating room such as bronchoalveolar lavage, guided tracheal intubation, and tracheotomy. Whether SFBs can be used in more clinical operations needs further study. At present, there is no comparative study between SFB and RFB in the aspects of biopsy and interventional therapy. This study aimed to explore whether SFB can perform complex bronchoscopic procedures such as transbronchial biopsies just like RFB.

## Materials and methods

### Subjects and study design

**Fig. 1:**
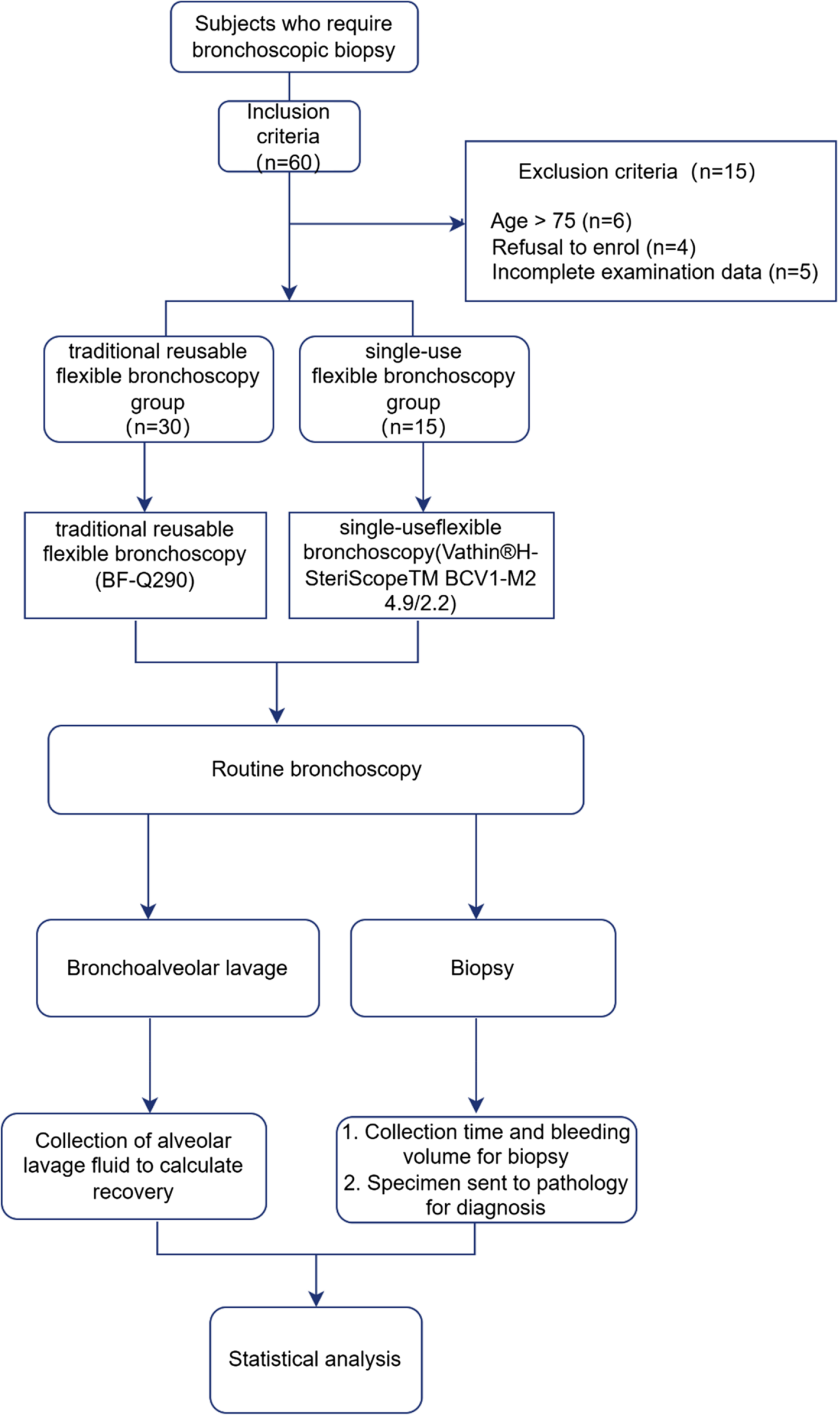
study flowchart

This single-center prospective controlled trial included 45 patients (aged 41 ~ 74 years) who needed bronchoscopic biopsy (Fig. 1). Subjects were recruited from January 2022 to December 2022 from the Third Xiangya Hospital of Central South University. Inclusion criteria: ① age range 18–75 years; ② patients underwent bronchoscopy in our hospital, and all signed an informed consent form; ③ lung CT showed block shadow, pulmonary atelectasis, obstructive pneumonia, and suspected lung cancer. Exclusion criteria were those with contraindications for bronchoscopy [16, 17]. The Ethics Committee of the Third Xiangya Hospital of Central South University approved this study (approval number: R22029). Then it passed the China Clinical Trial Center registration on 20/02/2023 and obtained the trial registration number (ChiCTR2300068434).

### Equipment

The study was conducted at the Third Xiangya Hospital of Central South University. The operators performed routine bronchoscopy, bronchoalveolar lavage, and biopsy using SFBs (Vathin®H-SteriScopeTM BCV1-M2 4.9/2.2) and RFBs (BF-Q290, Olympus Corporation) respectively. The comparison of the main parameters of the two types of bronchoscopes is described in Table 1.

**Table 1:** Features of RFB and SFB

	**RFB**	**SFB**
Insertion Tube OD (mm)	4.9	4.9
Distal and OD (mm)	4.8	5.0
Channel ID (mm)	2.0	2.2
Bending (up/down)	180–210°/130°	210°/210°
Rotary Function (left/right)	120°/120°	90°/90°
Working Length (mm)	600	600
Depth of Field (mm)	2–100	3–50
Field of View	120°	110° ± 5°
Risk of cross infection	Yes	No
Portability	No	Yes
Maintenance Cost	Expensive	Zero

*OD* Outer diameter

*ID* Inner diameter

### Methods

#### Routine bronchoscopy

Patients did not take food or water 6 h before the operation [17]. Patients were again informed of the risks involved and signed an informed consent form before the examination [18, 19]. During the examination, the patients were given oxygen inhalation (oxygen saturation > 90%) and their vital signs were monitored [17]. Each group underwent routine bronchoscopy with RFB or SFB. Before insertion of the bronchoscope, the patient's nasopharynx and oropharynx were locally anaesthetized with lidocaine, and then the bronchoscope was skillfully inserted transnasally. As the bronchoscope was inserted, the nasopharynx, oropharynx, larynx, vocal cords, ridge and bronchi were gradually examined, during which lidocaine was sprayed for local anesthesia [17]. The bronchus on the healthy side is usually examined first, followed by the bronchus on the affected side. The routine examination time for each group is recorded separately at the end of the examination (Fig. 1).

#### Bronchoalveolar lavage

The operator fixed the front end of the bronchoscope to the opening of the lesion, injected 20 ml warm saline solution into the lung segment through the working channel, and then gently aspirated the liquid (The pressure < 100 mmHg) into a sterile bottle for 3 times, The total volume of lavage was 60 ml, and the amount recovered was recorded [20-22].

#### Bronchoscopic biopsy

First, we observed and recorded the size and location of the lesion via bronchoscopy and defined the biopsy target. Biopsy forceps (ATE-QYQ-A-181050, Jiangsu Antel Medical Technology Corporation) was then inserted through the operating channel. We performed a total of five biopsies until a satisfactory specimen was obtained [23], then we recorded each patient’s biopsy time. The tissue specimens were fixed in 10% formalin and histopathology was examined.

#### Performance evaluation

After each procedure, the bronchoscope operator scored the performance of the SFBs. A score of 1 represents very poor, and 5 represents very well. The evaluation content included image quality (clarity), image brightness, eye comfort, viewing range, button maneuverability, interface icon layout, resistance to interference) of the bronchoscope mainframe, and the endoscope insertion process, fit with the tracheal tube, handle suction button maneuverability, suction function suction volume, handle biopsy cap ease of use (water and air injection), operating handle control(such as bending angle, bending circle diameter, force, resistance, the weight of the endoscope, compatibility with other surgical devices, and so on.

### Questionnaire survey

We designed a questionnaire to investigate and analyze the understanding of SFBs by different doctors. The questionnaire included 16 questions, such as the current knowledge of SFBs, the use of SFBs status, application prospects, and so on. Participants filled in the questionnaire through the “Questionnaire Star” small procedure.

### Statistical analysis

The measurement data were expressed as mean ± standard deviation (X ± S) and analyzed by a two-sample t-test. The counting data were expressed as a percentage (%) or constituent ratios, the χ^2^ test was used for statistical analysis to compare groups, and SPSS25.0 software was used for statistical analysis, *P* < 0.05 indicated the difference was statistically significant.

## Results

### Patients’ clinical characteristics

Forty-five patients (40 males), with a mean age of 63 years (range 41–74 years), were enrolled. CT scan of the lung shows a central type of lung cancer. The clinical characteristics of the population are shown in Table 2.

**Table 2:** Demographics

	**RFB (*n* = 30)**	**SFB (*n* = 15)**	**P**
Age (yr, mean ± SD)	62.12 ± 24.38	63.8 ± 16.9	0.263
Gender			
Males, n (%)	26(87%)	14(93%)	
Females, n(%)	4(13%)	1(7%)	
Smoking	22(73%)	11(73%)	
BMI(kg/m2,mean ± SD)	21.32 ± 3.17	22.19 ± 2.34	0.362
Pulmonary function (FEV1/ pred, mean ± SD)	68.34 ± 17.68	69.16 ± 11.77	0.874
Co-morbidities, n(%)			
COPD	6(20%)	2(13%)	
CHD	NA	1(7%)	
Diabetes	2(7%)	1(7%)	
Hypertension	4(13%)	2(13%)	
NSD	2(7%)	1(7%)	
Hepatic insufficiency	NA	2(13%)	

### Comparison of the routine examination time, recovery rate of bronchoalveolar lavage fluid(BALF), biopsy time, and positivity rate between RFB Group and SFB Group

**Fig. 2:**
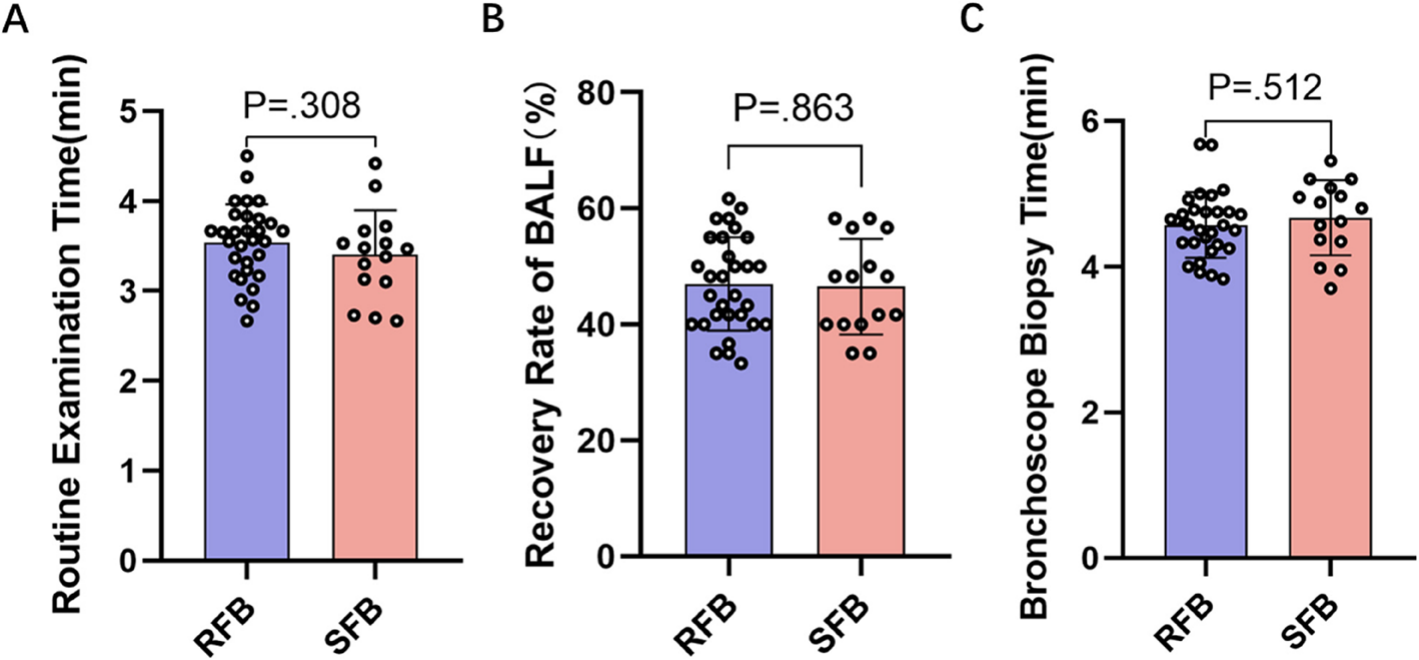
**A**: Comparison of Routine Examination Time between RFB vs SFB; **B**: Recovery Rate of BALF from RFB vs SFB; **C**: Bronchoscope Biopsy Time from RFB vs SFB

The routine examination time of SFB and RFB was 3.40 ± 0.50 min and 3.55 ± 0.42 min respectively. There was no significant difference between the two groups (*P* = 0.308) (Fig. 2A). The recovery rate of BALF was (46.56 ± 8.22) % in the SFBs group and (47.00 ± 8.07) % in the RFBs group, without a significant difference between the two groups(*P* = 0.863) (Fig. 2B).

Both groups experienced a small amount of bleeding during the biopsy, and no complications such as massive bleeding, pneumothorax, or arrhythmia were seen; there were no relevant complications at postoperative follow-up. The biopsy time was 4.67 ± 0.51 min in the SFBs group and 4.57 ± 0.45 min in the RFBs group, with no significant difference between the two groups (*P* = 0.512) (Fig. 2C). The positive biopsy rate was 100% in both groups, without significant difference (Table 3).

**Table 3:** Diagnostic rate of Bronchoscopy biopsy by RFB and SFB. n(%)

Pathological diagnosis of bronchoscopic biopsy	RFB	SFB	P
positive^a^	30(100%)	15(100%)	1.00
Squamous cell carcinoma	16(53%)	10(67%)	
Adenocarcinoma	4(13%)	1(7%)	
Small cell carcinoma	4(13%)	1(7%)	
A small round cell malignant tumor	4(13%)	1(7%)	
Malignant tumor^b^	2(7%)	1(7%)	
SMARCA4 deletion undifferentiated carcinoma	0(0%)	1(7%)	
negative	0(0%)	0(0%)	

^a^ using the χ^2^ test, Two groups of the biopsy positive rate do not exist difference

^b^Malignant tumor category is unknown

### Performance evaluation of SFBs

**Fig. 3:**
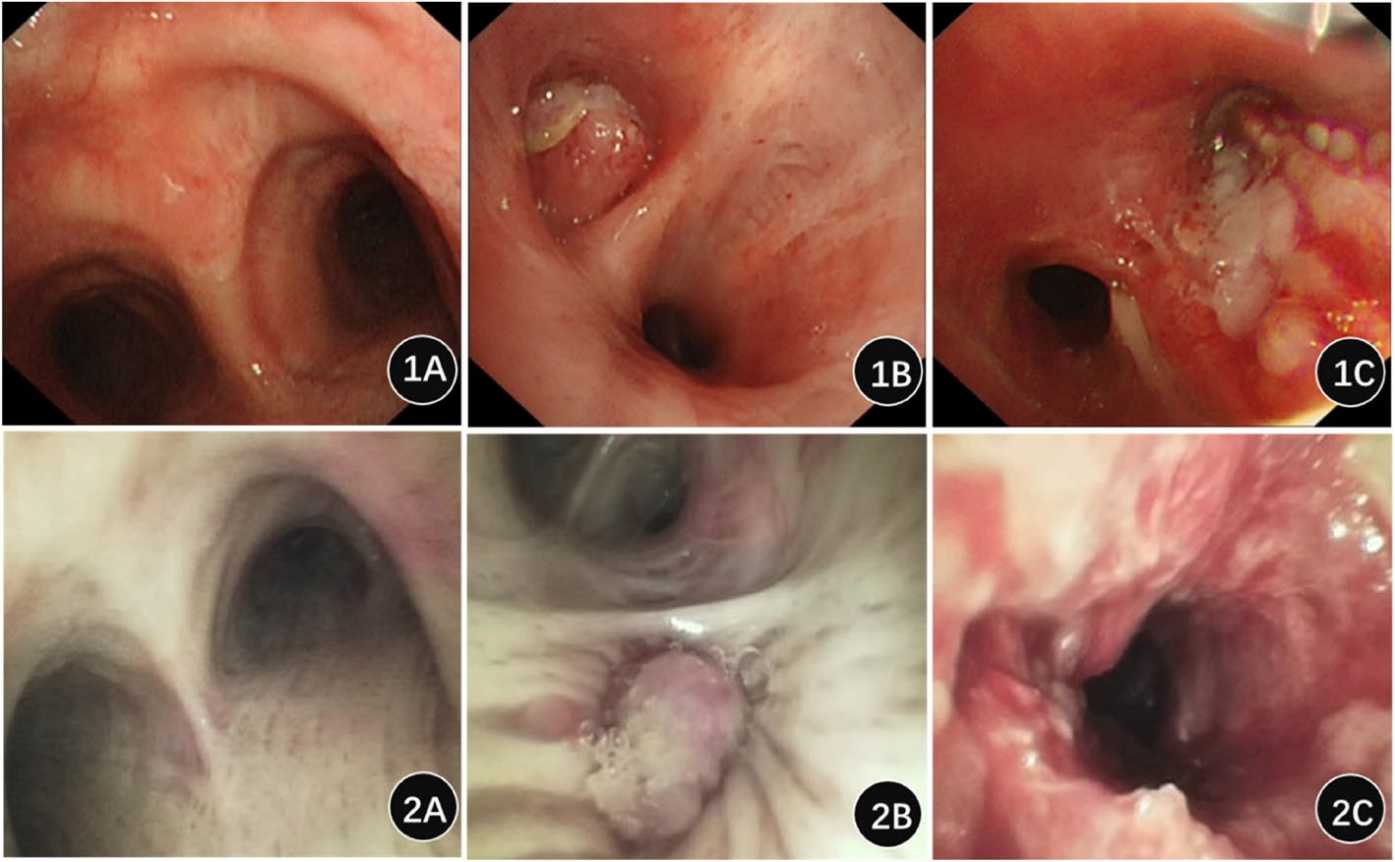
Comparison of imaging changes between RFB and SFB. Note: 1A-1C: Imaging changes of RFBs, 2A-2C: Imaging changes of SFBs

The performance of the SFBs was evaluated by a questionnaire from 9 operating physicians, and the results are shown in Table 4 and Table 5. In general, the operators were satisfied with SFBs. They considered it outstanding in portability (4.67 ± 0.5 points). The device insertion process (4.33 ± 0.5 points), observation Range, keystroke manipulation, Interface icon layout, and handle suction button operability (4.22 ± 0.44 points) were Good. However, the performance in terms of image clarity, brightness, and resistance to interference was average. Especially the image brightness score was the lowest(3.00 ± 0 points).As shown in Fig. 3, we can compare imaging changes between RFB and SFB. Figure 1A and 2A, respectively, present images of the normal ridge of RFB and SFB, and Figs. 1B-1C and 2B-C present images of different lesions in the bronchi lumen of RFB and SFB, respectively, including neoplastic occlusion and erosion. SFBs can display the image of the lesion very well.

**Table 4:** Performance Evaluation Scores of SFB host

Bronchoscope Operator	Insertion process	Compatibility with endotracheal intubation	Handle suction button operability	Attraction function attraction quantity	Handle biopsy cap Ease of use	Operating handle control	Endoscope weight	Compatibility with other surgical equipment
1	4	4	4	4	4	4	4	4
2	5	5	5	5	5	5	5	5
3	4	4	4	4	4	4	5	4
4	4	4	4	4	4	4	5	4
5	4	4	4	4	4	4	5	3
6	4	3	3	4	4	3	4	4
7	5	5	5	4	3	4	5	4
8	5	4	5	4	5	5	5	4
9	4	3	4	4	4	4	4	4
Mean ± SD	4.33 ± 0.0	4.00 ± 0.71	4.22 ± 0.70	4.11 ± 0.3	4.11 ± 0.60	4.11 ± 0.60	4.67 ± 0.50	4.00 ± 0.50

**Table 5:** Performance Evaluation Scores of SFB Endoscope

Bronchoscope Operator	Image Clarity	Image Brightness	Eye Comfort	Observation Range	Keystroke manipulation	Interface icon layout	Anti-interference ability
1	3	3	3	4	4	4	3
2	4	3	5	5	5	5	5
3	4	3	4	4	4	4	3
4	4	3	4	4	4	4	4
5	4	3	4	4	4	4	4
6	4	3	4	4	4	4	4
7	4	3	4	5	5	5	4
8	4	3	4	4	4	4	5
9	4	3	3	4	4	4	3
Mean ± SD	3.89 ± 0.33	3.00 ± 0	3.89 ± 0.6	4.22 ± 0.44	4.22 ± 0.44	4.22 ± 0.44	3.89 ± 0.78

### Questionnaire survey on the clinical application of SFBs

**Fig. 4:**
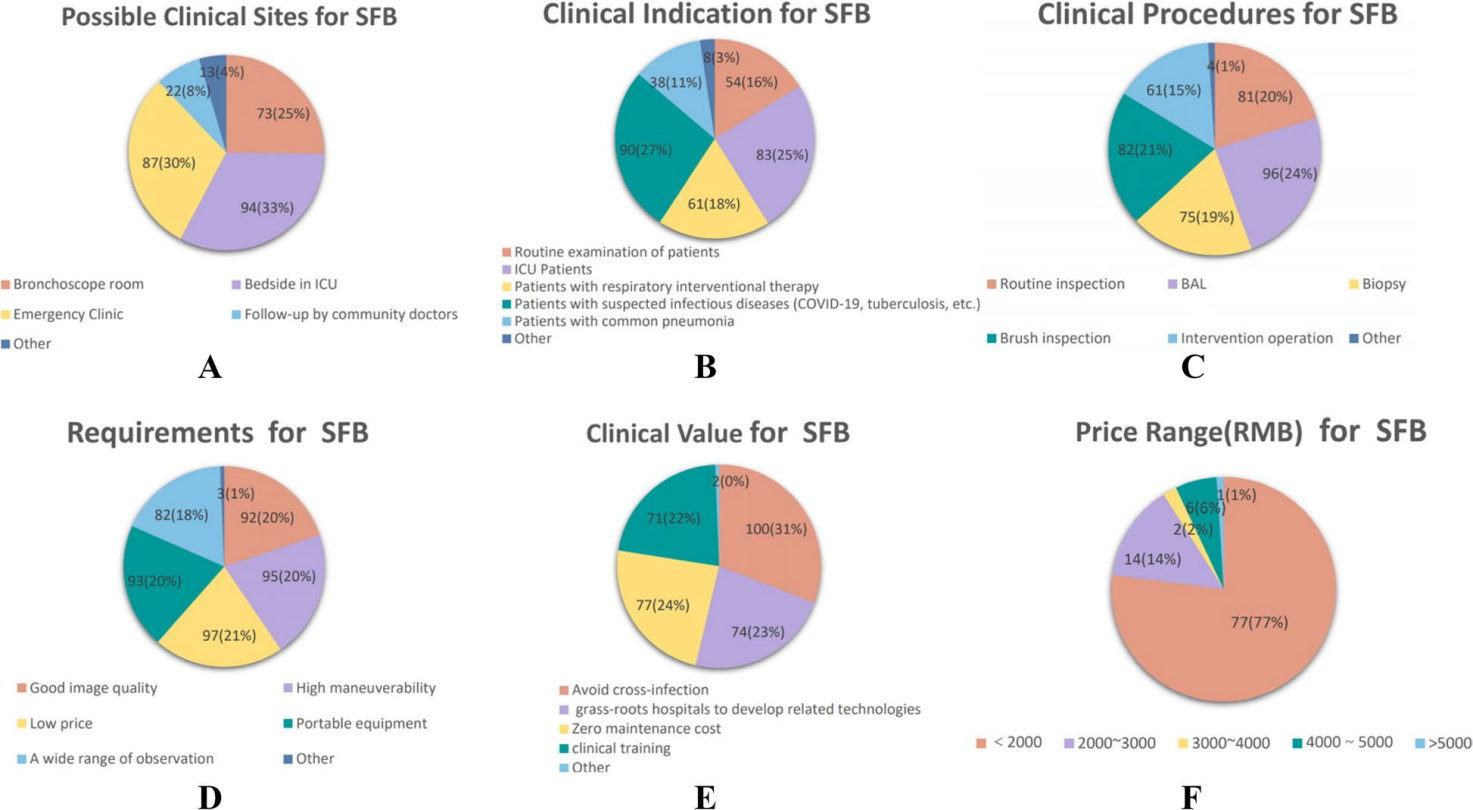
Questionnaire Survey Results of SFBs,n (%). Note: **A**, **B**,**C**,**D**,**E** in the questionnaire survey are multiple choice

A total of 100 physicians from different hospitals participated in this survey. Most of the participants were respiratory physicians(73) and some were ICU physicians(13). Participants had less knowledge and use of SFBs. The top three clinical sites for possible use of SFBs were bedside in ICU (94), emergency clinic (87), and bronchoscopy room (73) (Fig. 4A); In the case of SFBs, up to 90 patients were suspected infectious diseases (such as COVID-19, tuberculosis, etc.), followed by ICU patients (83),then patients with respiratory interventional therapy (61) (Fig. 4B); The clinical requirements for SFBs are bronchoalveolar lavage(BAL)(96), brush inspection (82), routine inspection (81), biopsy (75), and intervention (61) (Fig. 4C); The performance of requirements for SFBs is low price (97), high maneuverability (95), portable equipment (93), good image quality (92), etc.(Fig. 4D);The clinical value of SFBs is undoubtedly the avoidance of cross-infection(100), followed by zero maintenance cost (77), etc.(Fig. 4E); Finally, 77% participants only accepted the price of SFB below £238 (Fig. 4F).

## Discussion

Since the launch of the Ambu aScope in 2009, SFBs have undergone more than 10 years of continuous improvement from the original fibrescope to the electron bronchoscope [12]. The technology of the Complementary Metal Oxide Semiconductor (CMOS) [24], the core component of the image sensor, is also mature, which improves the problems such as poor image quality and low sensitivity [25]. Currently, SFBs have been used in ICU or perioperative settings, and operation is limited to BAL, guided tracheal intubation, tracheotomy, etc. In this study, we further explored more clinical applications of SFBs, such as transbronchial biopsy, and compared the performance between SFBs and RFBs.

In routine bronchoscopy, we found no significant differences in comparing their examination times. The recovery rate of qualified BALF should be more than 30% [22]. Our study found that both SFBs and RFBs could achieve satisfactory recovery rates, which was consistent with previous studies. Zaidi et al. found that SFBs obtained more excellent recovery of BALF than RFBs, and there was no significant difference in cell number and survival rate between the two groups [21]. Furthermore, in this study, SFBs were used in transbronchoscopic biopsy for the first time. There was no significant difference in biopsy time and positivity rate between SFBs and RFBs, which confirmed that SFBs could perform more bronchoscopic operations. All of the above confirmed that SFBs are non-inferior in routine bronchoscopy, BAL, biopsy, and so on.

Compared to RFBs, SFBs eliminate the risk of bronchoscopy-related infection. In the context of the COVID-19 global pandemic, several societies, including the American Association of Bronchology and Interventional Pulmonology (AABIP) and the Spanish Society of Pulmonology and Thoracic Surgery (SEPAR), recommended the use of SFBs in patients with suspected or confirmed COVID-19 infection, to reduce the spread of disease and protect healthcare staff [15]. In this study, we followed up with patients for two weeks after clinical operations with SFBs [26], and none of them had infection-related symptoms.

In terms of performance comparison, Liang et al. found that the YunSendo-R(SFBs) is superior to the Ambu aScope3 (SFBs) in terms of image clarity, color contrast, and illumination [27]. And it had similar vision and operability to the Olympus bronchoscope (RFBs). By investigating operator perceptions, Liu et al. found no significant difference in operability between the Vathin H-SteriScope and RFB [14]. However, Flandes et al. argued that 54.4% of operators considered that the image quality of the SFBs was worse than the RFBs [28]. In our study, the operators were generally satisfied with the SFBs after completing clinical operations, particularly in portability and lightness. However, the shortcomings of SFB are also obvious, in the image clarity, and lighting, which the operators said needs to be improved. To reduce the cost of the SFBs, CMOS is an essential component. However, compared to the charge-coupled device (CCD)[29, 30] used in RFBs, CMOS still has disadvantages such as poor image quality, low resolution, and low light sensitivity, which explains why RFBs do not perform as well as RFBs. In the future, the technique of SFBs should be improved to meet the clinical needs better.

In order to better understand the clinical needs, we designed a questionnaire. We found that SFBs are not yet widely used in China, and most doctors’ knowledge about SFBs is only obtained from literature or the internet. On the basis of avoiding cross-infection effectively, doctors demanded lower prices, better manipulation, and superior image quality of SFBs. Especially in price, 77% of those surveyed only accept SFBs less than £238. In the aspect of cost, detailed comparisons of RFB and SFB costs have varied between studies. For example, Tvede et al. [31] suggested that SFBs were not economically suitable for use. In contrast, Mouritsen et al. [8] analyzed micro-costs to find that the per-use cost of RFB is £29.20 higher than SFB; The cost-effectiveness analysis demonstrated a potential saving of £291.00 (additional treatment costs for bronchoscopic infections) could be achieved with SFB compared to RFB. In general, although the cost per use of SFB may be higher than RFB for patients, the total cost of SFB is reduced compared to RFB in terms of infection control and overall healthcare resource consumption, which could also be adjusted in the future through hospital fees or health insurance, and so on. In addition, Sohrt et al. [32] mention that the advantage of scale may also affect SFB prices, and in the future, the market competition among disposable bronchoscope manufacturers and technological advances may also drive down the cost of SFB.compared with RFBs, SFBs have low operation costs, low site requirements, and zero maintenance costs. We believed that SFBs will better meet the clinical needs with the continuous development of technology.

To summarize, our study confirmed that SFBs are non-inferior to RFBs in bronchoscopy, BAL, biopsy, and so on. It expands the new clinical application of SFBs and has certain clinical significance. This study has some limitations. Such as it is a single-center study with a small sample size. There is a potential sampling bias in this study.

In the future, more clinical applications of SFBs can be explored, such as peripheral lesion biopsy, interventional therapy, physician training, and so on. Technical requirements such as improving imaging quality and illumination are also the direction of future improvement of SFBs.

## Conclusions

The small sample size study initially hints SFBs may be non-inferior to RFBs in routine bronchoscopy, bronchoalveolar lavage, and biopsy. It is suggested that SFBs have a wider clinical application.

## Data Availability

The datasets used and/or analysed during the current study are available from the corresponding author (Shuzhen He/Lihua Xie) on reasonable request.

## Abbreviations

SFB: Single-use flexible bronchoscope
RFB: Reusable flexible bronchoscope
BALF: Bronchoalveolar lavage fluid
BAL: Bronchoalveolar lavage
ICU: Intensive care unit
ID: Inner diameter
OD: Outer diameter
BMI: Body mass index
FEV1: Forced expiratory volume in 1 s
COPD: Chronic obstructive pulmonary disease
CHD: Coronary heart disease
NSD: Nervous system disease
CMOS: Complementary metal oxide semiconductor
CCD: Charge-coupled device
